# Expression and Function of the Chemokine, CXCL13, and Its Receptor, CXCR5, in Aids-Associated Non-Hodgkin's Lymphoma

**DOI:** 10.1155/2010/164586

**Published:** 2010-08-25

**Authors:** Daniel P. Widney, Dorina Gui, Laura M. Popoviciu, Jonathan W. Said, Elizabeth C. Breen, Xin Huang, Christina M. R. Kitchen, Juan M. Alcantar, Jeffrey B. Smith, Roger Detels, Otoniel Martínez-Maza

**Affiliations:** ^1^Department of Obstetrics & Gynecology, David Geffen School of Medicine at UCLA, CA 90095-1740, USA; ^2^Department of Pathology and Laboratory Medicine, David Geffen School of Medicine at UCLA, CA 90095-1732, USA; ^3^UCLA AIDS Institute and Jonsson Comprehensive Cancer Center, University of California, Los Angeles, CA 90095, USA; ^4^Department of Psychiatry & Biobehavioral Sciences, David Geffen School of Medicine at UCLA, CA 90095-6967, USA; ^5^Los Angeles Center of the Multicenter AIDS Cohort Study (MACS), University of California, Los Angeles, CA 90095, USA; ^6^Department of Biostatistics, School of Public Health, University of California, Los Angeles, CA 90095, USA; ^7^Department of Pediatrics, David Geffen School of Medicine at UCLA, CA 90095-1752, USA; ^8^Department of Epidemiology, School of Public Health, University of California, Los Angeles, CA 90095, USA; ^9^Department of Microbiology, Immunology & Molecular Genetics, David Geffen School of Medicine at UCLA, CA 90095-7364, USA; ^10^UCLA AIDS Institute, BSRB Room 173, 615 Charles Young Drive South, Los Angeles, CA 90095-7363, USA

## Abstract

*Background*. The homeostatic chemokine, CXCL13 (BLC, BCA-1), helps direct the recirculation of mature, resting B cells, which express its receptor, CXCR5. CXCL13/CXCR5 are expressed, and may play a role, in some non-AIDS-associated B cell tumors. *Objective*. To determine if CXCL13/CXCR5 are associated with AIDS-related non-Hodgkin's lymphoma (AIDS-NHL). *Methods*. Serum CXCL13 levels were measured by ELISA in 46 subjects who developed AIDS-NHL in the Multicenter AIDS Cohort Study and in controls. The expression or function of CXCL13 and CXCR5 was examined on primary AIDS-NHL specimens or AIDS-NHL cell lines. *Results*. Serum CXCL13 levels were significantly elevated in the AIDS-NHL group compared to controls. All primary AIDS-NHL specimens showed CXCR5 expression and most also showed CXCL13 expression. AIDS-NHL cell lines expressed CXCR5 and showed chemotaxis towards CXCL13. *Conclusions*. CXCL13/CXCR5 are expressed in AIDS-NHL and could potentially be involved in its biology. CXCL13 may have potential as a biomarker for AIDS-NHL.

## 1. Introduction

HIV infection is associated with a greatly increased risk of non-Hodgkin's B cell lymphoma (NHL) [1–3]. The increased incidence of NHL in HIV-infected persons is believed to be an end result of excess B cell activation that is seen during HIV infection, as well as of loss of immunoregulatory control of Epstein-Barr virus (EBV) infection [1]. AIDS-NHL tumors are generally of high grade and can be broadly placed into one of several subtypes, which include Burkitt lymphoma (AIDS-BL), diffuse large B cell lymphoma (AIDS-DLBCL), and primary CNS lymphoma [4, 5]. Levels of B cell-stimulatory cytokines, such as IL-6, are known to be elevated during HIV infection and are further elevated in those HIV(+) persons who go on to develop NHL [1, 6–8]. Thus, cytokines may be important in the development and growth of these tumors. 

Although it has been shown that cytokines probably play an important role in the biology of AIDS-NHL, little work has been done on chemokines, cytokines that are most known for their ability to direct chemotaxis of immune cells [9]. In recent years, it has been shown that chemokines can play important roles in a variety of cancers, including cancers of the breast, prostate, lung, pancreas, liver, and skin, colorectal cancer, and cancers of the immune system, including some lymphomas [10–18]. Chemokines can potentially promote tumor progression by a number of means, including by directly promoting tumor cell growth, by inhibiting tumor cell apoptosis, and by promoting tumor cell migration and metastasis [10–12, 19]. 

Although, in general, not much is known about the role that chemokines could be playing in AIDS-NHL, several studies are extant in the literature that cover aspects of this issue. Specifically, Rabkin et al. have reported that HIV(+) subjects who are at least heterozygous for a genetic deletion in the chemokine receptor, CCR5, are at significantly decreased risk for developing NHL. Conversely, HIV(+) individuals who carry particular polymorphisms in the gene for the chemokine, SDF-1, have a substantially increased risk of developing NHL [20]. Sei et al. have reported increased expression of SDF-1 in children who had AIDS-NHL [21]. Sharma et al. reported that several AIDS-NHL cell lines secreted the chemokines, IL-8, IL-16, and MIP-1*α* [22–24]. Some cell lines also expressed the receptor for MIP-1*α*, indicating the potential for autocrine interactions [24]. Thus, it seems likely that chemokines can play a role in the biology of AIDS-NHL. 

Recently, we reported that serum levels of the chemokine, CXCL13, are substantially elevated during HIV infection [25]. CXCL13 is a homeostatic chemokine that helps direct the normal trafficking of B cells in the body [26]. It is expressed by T follicular helper cells, dendritic cells, and stromal cells in B cell areas of secondary lymphoid tissue, and it chemoattracts mature, circulating, B cells, which express its receptor, CXCR5, into these zones [27–31]. B cells become activated in these zones if given appropriate help, and then may further differentiate to become antibody-producing cells [32]. Two other reports indicate that there are abnormalities in the CXCR5/CXCL13 system during HIV infection: first, Förster et al. reported some years ago that many recirculating mature B cells lose expression of CXCR5 during HIV infection; in contrast, these cells nearly uniformly express CXCR5 in healthy, uninfected, individuals [33]. Second, Cagigi et al. have recently shown that recirculating B cells may express CXCL13 in HIV-infected subjects, in contrast to B cells from healthy, uninfected, subjects, which are uniformly negative for expression of this molecule [34]. These observations raise the possibility that abnormalities in the CXCR5/CXCL13 system are contributing to the abnormalities that are seen in the B cell compartment during HIV infection, and thus could be involved in the genesis of AIDS-NHL. 

It should be noted that CXCR5 and/or CXCL13 have been shown to be associated with several non-AIDS-related B cell lymphomas, including extragastric lymphoma of mucosa-associated lymphoid tissue (MALT) and follicular lymphoma [13–17, 35]. In several of these cancers, tumor cells were shown to express functional CXCR5 and to migrate towards CXCL13 [17]. In some of these cancers, tumor cells were shown to express both CXCL13 and CXCR5, suggesting a possibility for autocrine interactions, or CXCL13 was shown to be expressed in tissue surrounding tumors, raising the possibility that it could be directing the metastasis of tumor cells [16, 17, 35, 36]. In this report, we define the expression and function of CXCL13 and CXCR5 in AIDS-NHL.

## 2. Materials and Methods

### 2.1. Subjects

Archival stored serum specimens were obtained from the Los Angeles (UCLA) center of the Multicenter AIDS Cohort Study (MACS), an ongoing natural history study of HIV in adult homosexual men established in 1984 with 1637 original participants [37, 38]. In the MACS, participants are examined semiannually, and blood samples are archived at each visit. The Institutional Review Board at UCLA approved this study. Our study assessed CXCL13 levels in sera collected from four groups of adult homosexual men: Group I (AIDS-NHL) consisted of 46 subjects who developed AIDS-lymphoma. For each subject, archival serum was used from the closest available visit prior to lymphoma diagnosis. The mean time to diagnosis was 8.2 months (SD = 7.9). Of the 46 subjects in Group I, 15 subjects had AIDS-NHL of the Burkitt subtype, 29 had AIDS-NHL of the diffuse large B cell lymphoma (DLBCL) subtype, and 2 had AIDS-NHL of unspecified subtype. Group II (AIDS, non-lymphoma) consisted of 41 subjects with AIDS [39], but no reported malignancy. The mean CD4 T cell counts of Group I (199 cells/mm^3^, SD = 198) and Group II (182 cells/mm^3^, SD = 162) were similar. Group III subjects (*n* = 43) were HIV(+), but had no AIDS-defining condition and were selected to have CD4 T cell counts greater than 500 (mean = 684 cells/mm^3^, SD = 181). Group IV subjects (*n* = 40) were HIV seronegative. All subjects were naïve for highly active antiretroviral therapy (HAART).

### 2.2. Determination of Levels of CXCL13 and Other Markers

CXCL13 levels in sera, and in culture supernatants of cell lines, were determined by ELISA (R&D Systems, Minneapolis, MN; lower detection limit = 16 pg/mL). Levels of soluble CD44std (sCD44) in sera were determined by ELISA in earlier studies using the same sera as in the current study [40]; this data was combined with the CXCL13 data and used in correlational analyses.

### 2.3. Immunohistochemistry Studies

Tissue arrays containing formalin-fixed, paraffin-embedded sections from AIDS-lymphomas were obtained from the AIDS and Cancer Specimen Resource (ACSR) of the National Cancer Institute. These AIDS-lymphomas were from subjects other than those who provided the serum samples for the CXCL13 serum studies described above, since tumor specimens were not available for most AIDS-NHL cases in the MACS that were used in the serum studies. Conversely, prediagnosis serum specimens were not available for the cases obtained from the ACSR. Of the AIDS-NHL tumors represented in the tissue arrays, about 1/3 were located in lymph nodes, while the remaining 2/3 were of extranodal origin. 

 Initially, one set of array slides was stained with standard hematoxylin and eosin (H&E) stain to allow general visualization of tissues and tumors. Tumors/tumor cells were generally easily identified in the arrays. 

 For immunohistochemical detection of specific markers, sections were initially deparaffinized in xylene and rehydrated through a graded alcohol series. Slides were pretreated for antigen retrieval in a steamer in citrate buffer (10 mM, pH 6.0) for 25 minutes at 90°C (CXCR5), or heated in a pressure cooker in TRIS (tris(hydroxymethyl)aminomethane) buffer (pH 9.0) for 2 minutes at 115°C (CXCL13). Endogenous peroxidase activity was then quenched with 0.3% H_2_O_2_ in 70% methanol for 10 minutes. The wash buffer in this study was PBS (phospho-buffered saline); all antibodies were diluted in PBS + 1% bovine serum albumin and used at room temperature. 

For CXCR5, a rat antihuman CXCR5 antibody (clone RF8B2, R&D Systems) or IgG_2b_ isotype control (clone KLH/G2b-1-2, Southern Biotechnology Associates, Birmingham, AL) was used (2 *μ*g/mL, 2 hours). Next, rabbit antirat polyclonal antibody (DAKO, Carpinteria, CA) and then horseradish peroxidase (HRP)-conjugated antirabbit EnVision reagent (DAKO) were applied, and color was developed using Fast Red (Innogenex, San Ramon, CA). 

For CXCL13, a specific polyclonal goat IgG (R&D Systems) was used (10 *μ*g/mL, 30 minutes); the control was normal goat IgG (Santa Cruz Biotechnology, Santa Cruz, CA). Next, a biotinylated secondary antibody (Vectastain Elite ABC kit, Vector Laboratories, Burlingame, CA) was applied, followed by Vectastain Elite ABC reagent. Color was developed using diaminobenzidine (Sigma-Aldrich, St. Louis, MO).

Individual array sections were scored by a pathologist as follows: 0 = no staining, 1+ = <30% of tumor cells positive, 2+ = 30%–60% of cells positive, 3+ = >60% of cells positive.

### 2.4. Cell Lines

Two AIDS-NHL cell lines, 2F7 (Burkitt subtype) and R (diffuse large B cell lymphoma subtype), and the Epstein-Barr virus (EBV)-lymphoblastoid B cell line, 40102, were used in these studies [41–43]. All cell lines were cultured in standard media as previously described [42]. Both AIDS-NHL cell lines are positive for the Epstein-Barr virus (EBV) and are not infected with HIV [41–43].

### 2.5. Flow Cytometry

Surface CXCR5 expression on cell lines was examined by flow cytometry using a previously described indirect staining protocol [42]. The primary anti-CXCR5 antibody was Clone RF8B2 (rat IgG_2b_, BD Biosciences, San Diego, CA). The isotype control antibody was clone KLH/Gb-1-2 (rat IgG_2b_, Southern Biotechnology Associates, Inc., Birmingham, AL). The secondary antibody was phycoerythrin (PE)-conjugated goat anti-rat IgG (Jackson ImmunoResearch Laboratories, Inc., West Grove, PA). Flow cytometry was performed using a Becton Dickinson LSR machine. Data was analyzed using CellQuest Pro 5.1 software (Becton Dickinson).

### 2.6. Cell Migration Assays

Cell migration assays were conducted on the 2F7 and R AIDS-NHL cell lines using the methods described by Kitchen et al. [44]. Briefly, cells were fluorescently prelabeled by culturing them for 30 minutes at 37° in the presence of 5 *μ*g/mL of Calcein-AM (Molecular Probes, Eugene, OR). Cells were then placed in RPMI 1610 medium without phenol red (Irvine Scientific, Irvine, CA) at a concentration of 3.6 × 10^6^ cells/mL. For determination of chemotaxis, cells (90,000; 25 *μ*L) were then loaded on top of the filter of ChemoTX chemotaxis plates (NeuroProbe, Gaithersburg, MD; filter pore size = 8-*μ*m). Recombinant human CXCL13 (R&D Systems) was then loaded into the bottom well, and plates were incubated for 2 hours at 37°C. Plate fluorescence was read using a Beckman Coulter (Fullerton, CA) D7X880 Multimode Detector (bottom read position; excitation, 485 nm; emission, 530 nm). A range of 0 (media only control) to 1000 ng/mL of CXCL13 was tested. Six to twelve replicate wells were used for each condition, and each experiment was performed at least three times. As an additional control, CXCL13 was loaded into both the lower and the upper wells at some locations on the plates, to test for chemokinesis. 

### 2.7. Statistical Analysis

Statistical analysis was performed using the SAS system, version 9.1 (SAS Institute, Cary, NC). In the study of serum CXCL13 levels, a moderate number of subjects had values below the lower limit of detection of the ELISA used (16 pg/mL). Therefore, the significance of differences in serum CXCL13 levels between groups was determined using a variant of the two-sample *t* test that accounted for left-censored values (i.e., values below the limit of detection of the assay), as previously detailed by us in an earlier publication [25]. As we noted in that previous reference, values less than 16 pg/mL were considered left-censored. We assumed that CXCL13 levels were distributed log-normal and used the method of maximum likelihood to estimate means and variance components of interest [45]. Although analysis was performed on the logarithmic scale, for ease of interpretation, means and standard deviations (SD) for CXCL13 are reported on the original scale using the delta method. A two-sample *z* test that accounted for the censoring was used to test for significance. 

The degree of association between serum CXCL13 and serum sCD44 was quantified using a bivariate linear regression analysis that again accounted for the left-censoring of CXCL13. Pearson correlation coefficients are used to report results of the regression analysis. For migration (chemotaxis) assays, differences between conditions were analyzed using the Wilcoxon rank sum test, as previously described [44].

## 3. Results

### 3.1. Serum CXCL13 Levels Are Elevated Prior to the Development of AIDS-NHL

**Figure 1:**
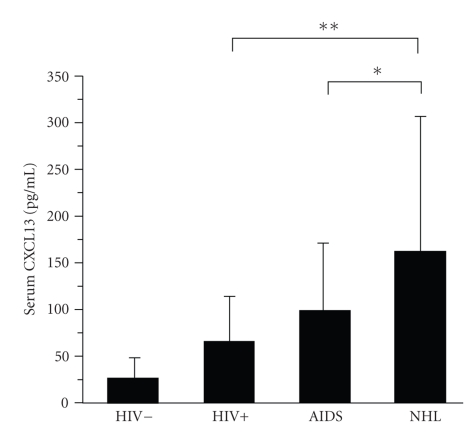
Serum CXCL13 levels are elevated prior to development of AIDS-NHL. Serum from each person in the AIDS-NHL group (*n* = 46) was obtained for one time point in the 2.5 years prior to the development of lymphoma; subjects in the AIDS control group (*n* = 41) had AIDS, but no malignancy. Subjects in the HIV(+) group (*n* = 43) were HIV-seropositive, non-AIDS, and had CD4 T cell numbers >500 cells/mm^3^. Serum CXCL13 levels were determined by ELISA. Subjects in the HIV(−) group (*n* = 40) were HIV-seronegative. Analysis was performed on the logarithmic scale, but we report means and standard deviations for CXCL13 on the original scale using the delta method, as previously reported [25]. Bars represent mean values; error bars represent standard deviations, (*P<  .05, and **P<  .001). Although not indicated in the figure, all other comparisons (HIV+ versus HIV−, AIDS versus HIV+, HIV− versus AIDS, and AIDS-NHL versus HIV−) were also significant, *P* < .05.

In these studies, we examined CXCL13 levels in AIDS-NHL subjects, using serum obtained at a mean time of 8.2 months prior to diagnosis. The mean CXCL13 level seen in the AIDS-NHL group (158 pg/mL, SD = 153) was ~50% higher than in the AIDS control group (98.4 pg/mL, SD = 70.9, *P* = .02, Figure 1). Furthermore, CXCL13 levels correlated with sCD44 levels in the AIDS-NHL group (R = 0.31, *P* = .04), but not in the AIDS control group (R = 0.07; *P* = .7, data not shown); we previously showed that sCD44 levels are elevated prior to AIDS-NHL development [40]. CXCL13 levels in the AIDS-NHL group were also ~2.5× greater than levels in the HIV(+) group, and ~7× greater than levels in the HIV(−) group; these results were statistically significant (Figure 1). No significant difference in CXCL13 levels was noted when comparing the Burkitt's and diffuse large B cell lymphoma (DLBCL) subtypes in the AIDS-NHL group (not shown), although this may be a result of the relatively small number of subjects tested in this study.

### 3.2. All Primary AIDS-NHL Specimens Showed CXCR5 Expression, and Most Showed CXCL13 Expression

**Figure 2:**
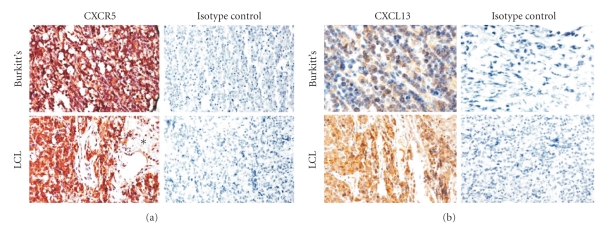
Representative expression of CXCR5 and CXCL13 protein in AIDS-NHL. Tissue arrays containing sections from numerous AIDS-NHLs were examined for expression of CXCR5 (a) or CXCL13 (b) by immunohistochemistry, as noted in Section 2. For CXCR5, an HRP/Fast Red system was used for color development (red); for CXCL13, Vectastain Elite ABC reagent and DAB were used (brown). Arrays were counterstained with hematoxylin. Sections representative of typical CXCR5 and CXCL13 staining patterns in AIDS-associated Burkitt lymphoma (AIDS-BL, indicated as “Burkitt's”) and AIDS-associated diffuse large B cell lymphoma (AIDS-DLBCL, indicated as “LCL”) are shown. Both AIDS-BL and AIDS-DLBCL show strong expression (3+) of CXCR5; AIDS-DLBCL shows strong expression (3+) of CXCL13, whereas AIDS-BL shows more moderate expression (2+). The sections shown representing CXCR5 and CXCL13 expression in AIDS-DLBCL came from the same tumor, an AIDS-DLBCL in the maxillary sinus. Normal sinus tissue (∗) is unstained. For each tumor section stained for CXCR5 or CXCL13 expression, a negative control using an isotype-specific, non-cross-reactive, antibody is shown on the right. All sections are shown at x100 original magnification, except that the Burkitt lymphoma in panel B is shown at x200 original magnification. Pictures were taken using an Olympus DP11 camera attached to an Olympus BX51 bright field microscope, and recorded on a Smart Media digital card. The 10x and 20x objective lenses (UPlan Apo, Japan) had apertures of 0.40 and 0.70, respectively. Pictures were edited for publication using Adobe Photoshop 6.0 and Canvas 5.0.3 (ACD Systems of America, Inc.).

For these studies, we examined CXCR5 and CXCL13 expression in tissue arrays containing sections of primary specimens of AIDS-NHL from different subjects, using immunohistochemistry. It was not technically feasible to examine both markers simultaneously using double staining. We therefore evaluated each marker using separate array slides containing sections from the same tumors. All AIDS-NHL tumors showed some degree of CXCR5 expression, and most (22/24) expressed CXCL13. Figure 2 shows representative staining for the two major AIDS-NHL subtypes, Burkitt lymphoma (BL), and diffuse large cell lymphoma (DLBCL). For AIDS-BL, the mean score for CXCR5 expression (*n* = 9) was 1.7 (range = 1 to 3); the mean score for CXCL13 expression (*n* = 7) was 1.0 (range = 0 to 3; data not shown). AIDS-DLBCL showed higher expression of both CXCR5 and, particularly, CXCL13; the mean score for CXCR5 (*n* = 19) was 2.2 (range = 1 to 3); the mean score for CXCL13 (*n* = 17) was 1.9 (range = 1 to 3; not shown). For most specimens, most expression of CXCR5 and/or CXCL13 appeared to be on tumor cells themselves, as most tumors did not appear to have many infiltrating cells (not shown). Eleven tumors had high numbers of cells expressing CXCR5 (i.e., scoring 2+ or above), and high numbers of cells expressing CXCL13, suggesting that some tumor cells coexpressed both markers. Indeed, this was very likely the case for several of the tumors, as virtually 100% of cells expressed CXCR5, and virtually 100% expressed CXCL13 (not shown). Sections of noncancerous lymph nodes were also included on the arrays as controls—these typically showed definite, but relatively low, expression of both molecules (a score of about 1; not shown), which likely reflects the previously reported observation that CXCL13 and CXCR5 expression in normal lymph nodes is generally confined to cells located in or near germinal centers [27, 29, 34].

### 3.3. AIDS-NHL Cell Lines Showed Surface Expression of CXCR5, and Sometimes Also Secreted Low Levels of CXCL13

**Figure 3:**
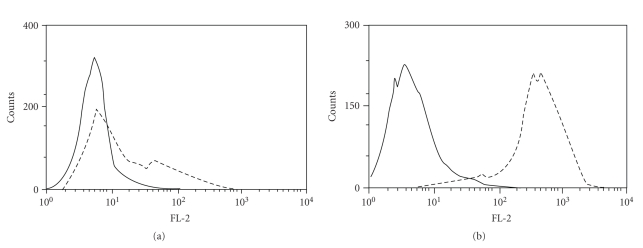
AIDS-NHL cell lines express CXCR5, as shown by flow cytometry. The AIDS-BL cell line, 2F7 (a), and the AIDS-DLBCL cell line, R (b), were stained for CXCR5 expression using an indirect staining protocol, as noted in Section 2. First, cells were stained with a rat IgG_2b_ anti-CXCR5 antibody (dotted lines). As a control, some cells were stained with a rat IgG_2b_ isotype control antibody (solid lines). Cells were then stained with a PE-conjugated goat anti-rat IgG secondary antibody, and examined by flow cytometry. During the flow cytometry acquisition stage, at least 5,000 cells/events were acquired per tube/condition. During analysis, dead cells were excluded using forward- and side-scatter.

In these studies, we examined expression of CXCR5 and CXCL13 on the AIDS-NHL cell lines, 2F7 (Burkitt subtype) and R (DLBCL subtype). Cell surface CXCR5 was detected using flow cytometry, and CXCL13 in cell culture supernatants was detected by ELISA. Both cell lines clearly expressed surface CXCR5; there was a large increase in mean fluorescence intensity (MFI) when cells were stained for CXCR5 compared to the isotype control (Figure 3). The two cell lines showed somewhat different patterns of CXCR5 expression, with cells of the R cell line uniformly showing high levels of CXCR5 expression. In contrast, cells of the 2F7 cell line showed a bimodal pattern of expression, with a large subpopulation expressing low levels of CXCR5, and a smaller subpopulation expressing higher levels of CXCR5 (Figure 3). Expression of CXCL13 was mixed, with culture supernatant of the R cell line not containing detectable levels of CXCL13 (<16 pg/mL; not shown). In contrast, culture supernatants of new cultures of 2F7 did not contain detectable levels of CXCL13, but culture supernatants of cultures of 2F7 that were several months old contained detectable CXCL13, albeit at low levels (19 ng/mL; data not shown). Culture supernatants of the EBV-lymphoblastoid cell line, 40102, which was used as a positive control, contained considerably higher levels of CXCL13 (42 pg/mL; not shown).

### 3.4. AIDS-NHL Cell Lines Showed Chemotaxis Towards CXCL13 In Vitro

**Figure 4:**
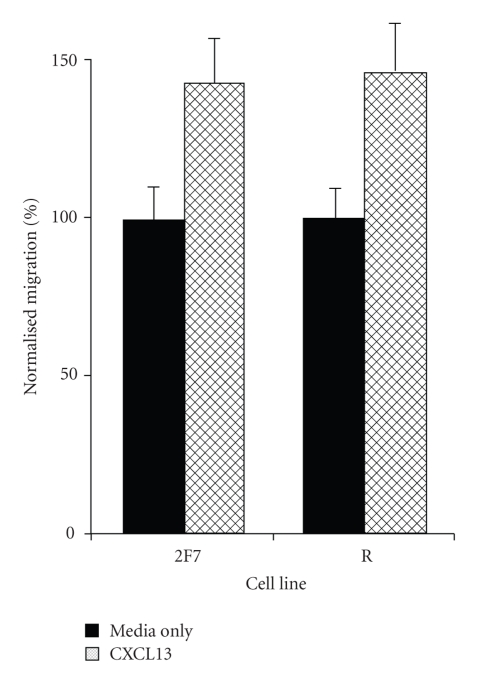
AIDS-NHL cell lines demonstrate chemotaxis towards CXCL13 in vitro. As noted in Section 2, cells of the AIDS-NHL cell lines, 2F7 (AIDS-BL) or R (AIDS-DLBCL), were first prelabeled with Calcein-AM, and then placed on top of 96-well chemotaxis chambers and permitted to migrate for 2 hours in response to CXCL13 or media alone placed in the lower wells. To create a standard curve, preset numbers of cells were added to some wells. Fluorescence in the bottom well was read in a plate reader, and this data was converted to number of cells migrated using the standard curve. As noted in Section 3, 100 ng/mL of CXCL13 was used for the 2F7 cell line, and 50 ng/mL was used for the R cell line. Each experiment was replicated at least 3 times, and, in each experiment, 6–12 replicate wells were used for each experimental condition. The figure shows data from a representative experiment for each cell line. Results are shown as average percent of cells migrating compared to the media only controls (which are set at 100%). Solid bars show results for media alone controls; hatched bars show results after addition of CXCL13. Bars represent the SEM.

The ability of the AIDS-NHL cell lines to respond to CXCL13 was examined using standard migration assays. Both cell lines demonstrated statistically significant (*P* < .05) migration towards CXCL13 (Figure 4). For the 2F7 cell line, 100 ng/mL of CXCL13 was optimal; 50 ng/mL was optimal for the R cell line. At its optimal concentration of CXCL13, each cell line always showed increased migration in each of 3-4 replicate experiments compared to the media only (spontaneous migration) control; the percent increase in cells migrated ranged from ~30% to ~90%. For both cell lines, the addition of CXCL13 to the upper well abrogated the response, indicating that chemotaxis, as opposed to chemokinesis, was occurring (not shown).

## 4. Discussion

These studies clearly demonstrate that there is an association between CXCL13 expression and AIDS-NHL. First, in the serum studies, serum CXCL13 levels were significantly elevated prior to lymphoma diagnosis in the AIDS-NHL group, compared to the AIDS control group (Figure 1). CXCL13 levels in the AIDS-NHL group were even more highly elevated when compared to the HIV(+)/non-AIDS and HIV-seronegative groups. These results suggest that assessment of CXCL13 levels could potentially be useful in the detection and diagnosis of AIDS-NHL. Elevated serum CXCL13 levels have recently been reported in other cancers, including B-cell chronic lymphocytic leukemia [46]. Second, many primary AIDS-NHL specimens, of both the Burkitt and DLBCL subtypes, showed expression of CXCL13 (Figure 2).Third, as noted in (Section 3), the AIDS-Burkitt cell line, 2F7, expressed low levels of CXCL13 after it had been growing in culture for several months (not shown). Thus, these combined data indicate that CXCL13 is frequently expressed in AIDS-NHL. These results are not dissimilar to results obtained in studies on several types of B cell lymphoma not associated with HIV infection, including non-AIDS-related primary central nervous system lymphoma (PCNSL) [15, 16, 35, 36]. The frequent expression of CXCL13 in the AIDS-NHL tumor specimens (Section 3, Figure 2) raises the possibility that the developing tumors themselves are a source of the elevated serum CXCL13 levels seen preceding diagnosis in the AIDS-NHL group (Figure 1). If so, CXCL13 could potentially prove to be a useful biomarker for early detection of AIDS-NHL, for determining burden of disease, and for determining prognosis following chemotherapy.

The receptor for CXCL13, CXCR5, appears to be commonly expressed in AIDS-NHL, as well. In the immunohistochemistry studies, all AIDS-NHL specimens showed expression of CXCR5 (Figure 2), as did both AIDS-NHL cell lines (Figure 3). Additionally, the CXCR5 on the AIDS-NHL cell lines appeared to be functional, as both cell lines showed chemotaxis towards CXCL13 (Figure 4). Again, these results are not dissimilar to other previously reported results for several types of B cell lymphoma not associated with HIV infection [13–17, 35, 36]. The fact that the optimal amount of CXCL13 in chemotaxis studies on the R cell line (50 ng/mL; Section 3) was lower than the optimal amount for the 2F7 cell line (100 ng/mL; Section 3) is consistent with the results in Figure 3, which indicate that the R cell line expresses higher levels of CXCR5 than the 2F7 cell line. 

Although our studies show that both CXCL13 and CXCR5 are commonly expressed in AIDS-NHL, future studies will be needed to determine more exactly what roles these molecules may be playing in these cancers. Several mechanisms, which are not necessarily mutually exclusive, appear possible: first, it seems plausible that CXCR5 and CXCL13 could be playing a role in the initial development of AIDS-NHL. As noted in Section 1, serum CXCL13 levels appear to be elevated during HIV infection, and at least some circulating B cells appear to (abnormally) express CXCL13, and to lose expression of CXCR5 [25, 33, 34]. As CXCL13/CXCR5 are important in guiding recirculating B cells into B cell zones of secondary lymphoid tissues [26, 27], if expression of these molecules is disturbed, it is possible that B cells will not home normally to these tissues. This could lead to inappropriate homing to other tissues and/or inappropriate activation, promoting lymphomagenesis. In support of this possibility is the fact that a large proportion of AIDS-associated lymphomas are extranodal, in contrast to B cell lymphomas not associated with HIV infection [47]. It could be that the continued expression of CXCL13/CXCR5 in developed AIDS-NHLs, as shown in the current studies, is a reflection of processes that occurred during lymphomagenesis. While these molecules might continue to have an effect on AIDS-NHL biology, it is also possible that their expression is merely an epiphenomenon that is reflective of the earlier role they played in the initial development of these tumors.

A second possibility is that the CXCL13 and/or CXCR5 could be directly promoting AIDS-NHL tumor growth, or performing some other tumor-promoting functions, such as inhibiting apoptosis or enhancing angiogenesis. Other chemokines, such as SDF-1, have been shown to directly induce tumor cell growth in non-AIDS-related cancers [11, 12], and it is possible that CXCL13 could be doing so, also. This promotion of cell growth could be coming from high levels of circulating CXCL13 produced elsewhere in the body, or from CXCL13 produced locally to the tumor, either by tumor cells themselves or by local nontumor cells. Our studies do suggest that autocrine interactions could be occurring in AIDS-NHL, as some tumor cells appeared to express both CXCR5 and CXCL13 in both the immunohistochemistry and AIDS-NHL cell line studies (Section 3). 

Third, it seems possible that CXCL13 could be directing movement and/or metastasis of AIDS-NHL cells in the body. As noted earlier (Figure 4), both AIDS-NHL cell lines used in our studies demonstrated chemotaxis towards CXCL13. Although CXCL13 has often described as being produced in secondary lymphoid tissues, it is now clear that it can be produced in other tissues as well, including brain, liver, and lung, particularly in the context of inflammation [48–50]. Perhaps CXL13 produced in such organs chemoattracts AIDS-NHL tumor cells to metastasize to these locations. 

It should be noted that at least aspects of these potential mechanisms involving CXCR5 and CXCL13 could be operating in some non-AIDS-related B cell lymphomas, as well, given the similarities noted above between the expression/function of these molecules in AIDS-NHL in the current study, and their previously reported expression/function in several types of non-AIDS-related B cell lymphoma [13–17, 35, 36]. However, it also seems possible that some of these mechanisms could prove to be unique to AIDS-NHL, given our previous finding that HIV infection is associated with an overproduction of CXCL13, even in subjects who do not develop lymphoma [25]. Such an overproduction of CXCL13 may not necessarily be occurring prior to the initiation of tumor development in otherwise healthy subjects who develop B cell lymphoma outside of the context of HIV infection. Thus, further study of CXCR5 and CXCL13 expression and function in AIDS-NHL, and in non-AIDS-associated NHL, could ultimately reveal some unique differences between the pathogenesis of AIDS-related and non-AIDS-related B cell lymphomas. 

In summary, we have demonstrated an association between expression of the chemokine, CXCL13, and its receptor, CXCR5, and AIDS-NHL. CXCL13 has potential as a biomarker for AIDS-NHL. Future studies will be needed to more fully determine the feasibility of using CXCL13 as a biomarker for AIDS-NHL, as well as to define the function of CXCL13 and CXCR5 in the pathogenesis of AIDS-NHL.
